# Drug-induced tooth discoloration: An analysis of the US food and drug administration adverse event reporting system

**DOI:** 10.3389/fphar.2023.1161728

**Published:** 2023-04-13

**Authors:** Jun Wang, Dongna Zou, Yuchao Li, Pingping Liu, Chenyu Guo

**Affiliations:** ^1^ Department of Pharmacy, Shandong Provincial Hospital Affiliated to Shandong First Medical University, Jinan, China; ^2^ Department of Medical Ultrasound, Shandong Provincial Hospital Affiliated to Shandong First Medical University, Jinan, China; ^3^ Department of Cardiology, Yantai Yuhuangding Hospital Affiliated to Qingdao University, Yantai, China; ^4^ Department of Pharmacy, Yantai Yuhuangding Hospital Affiliated to Qingdao University, Yantai, China

**Keywords:** drugs, tooth discoloration, FAERS, disproportionality analyses, data mining

## Abstract

**Background:** Certain drugs can cause intrinsic or extrinsic tooth discoloration, which is not only a clinical issue but also an esthetic problem. However, limited investigations have focused on drug-induced tooth discoloration. The present work aimed to determine the drugs causing tooth discoloration and to estimate their risks of causing tooth discoloration.

**Methods:** An observational, retrospective, and pharmacovigilance analysis was conducted, in which we extracted adverse event (AE) reports involving tooth discoloration by using the data of the US Food and Drug Administration’s Adverse Event Reporting System (FAERS) from the first quarter (Q1) of 2004 to the third quarter (Q3) of 2021. Disproportionality analyses were performed to examine risk signals for tooth discoloration and determine the drugs inducing tooth discoloration.

**Results:** Based on predefined inclusion criteria, 1188 AE reports involving 302 suspected drugs were identified. After data mining, 25 drugs generated positive risk signals for tooth discoloration, of which 10 were anti-infectives for systemic use. The top reported drug was tetracycline (*n* = 106), followed by salmeterol and fluticasone (*n* = 68), amoxicillin (*n* = 60), chlorhexidine (*n* = 54), and nicotine (*n* = 52). Cetylpyridinium (PRR = 472.2, ROR = 502.5), tetracycline (PRR = 220.4, ROR = 277), stannous fluoride (PRR = 254.3, ROR = 262.8), hydrogen peroxide (PRR = 240.0, ROR = 247.6), and chlorhexidine (PRR = 107.0, ROR = 108.4) showed stronger associations with tooth discoloration than the remaining drugs. Of 625 AE reports involving 25 drugs with positive risk signals, tooth discoloration was mostly reported in patients aged 45–64 (*n* = 110) and ≤18 (*n* = 95), and 29.4% (192/652) of the reports recorded serious outcomes.

**Conclusion:** This study revealed that certain drugs are significantly associated with tooth discoloration. Caution should be exercised when using these drugs, especially during pregnancy and early childhood.

## Introduction

With the continuous improvement of living standards, increasing attention is paid to oral health and the esthetic demand of keeping teeth white and shiny is gradually rising. The color of a tooth is affected by its inherent color and stains (Joiner and Luo, 2017). The inherent color of the tooth is primarily determined by dentine color and is affected by intrinsic and extrinsic factors (Watts and Addy, 2001).

Depending on the location of the stains, tooth discoloration can be classified into two types: intrinsic and extrinsic. Intrinsic tooth discoloration is usually caused by abnormal tooth development or stains deposited in the enamel of dentine during development, which is hard to remove. Extrinsic tooth discoloration usually occurs on the surface of the tooth, which may be removed by scaling and polishing. There are various factors inducing tooth discoloration, comprising intrinsic factors (e.g., some drugs and metabolic diseases) and extrinsic factors (e.g., wine, coffee, tobacco, and mouth rinses) (Kahler, 2022). Certain drugs can cause tooth discoloration after administration, among which tetracycline is the most characterized (Sánchez et al., 2004). However, drug-induced tooth discoloration is generally difficult to recognize and unfamiliar to clinicians. Therefore, it is necessary to identify drugs causing tooth discoloration as well as the appropriate population and timing of taking these medications. Unfortunately, no study has focused on comprehensively identifying the drugs associated with tooth discoloration, especially from the perspective of a representative population-based AE database.

The Food and Drug Administration Adverse Event Reporting System (FAERS) constitutes a publicly available and spontaneous reporting database containing AE reports, medication error reports, and product quality complaints resulting in AEs. The FAERS supports the FDA’s post-marketing safety surveillance program for drugs and therapeutic biologics (Hu et al., 2020). Herein, we retrieved AE reports involving tooth discoloration from the FAERS database. Disproportionality analyses were then performed to examine risk signals for tooth discoloration and to determine drugs causing tooth discoloration.

## Materials and methods

### Data source

This retrospective pharmacovigilance analysis was carried out with data in the FAERS database. In the current study, AE reports were extracted by using OpenVigil 2.1, an open web-based pharmacovigilance data extraction, cleaning, mining, and analysis tool used to examine the FAERS (Böhm et al., 2012).

### Identification of AE reports and drugs

The informatic structure of the FAERS system follows the international safety reporting guidance of the International Conference on Harmonisation (ICH). AEs are coded based on the Medical Dictionary for Regulatory Activities (MedDRA) terminology. MedDRA’s hierarchical structure includes system organ class (SOC), high level group term (HLGT), high level term (HLT), preferred term (PT), and lowest level term (LLT). Among them, PTs are single medical concepts, not a grouping (Mozzicato, 2009).

**FIGURE 1:**
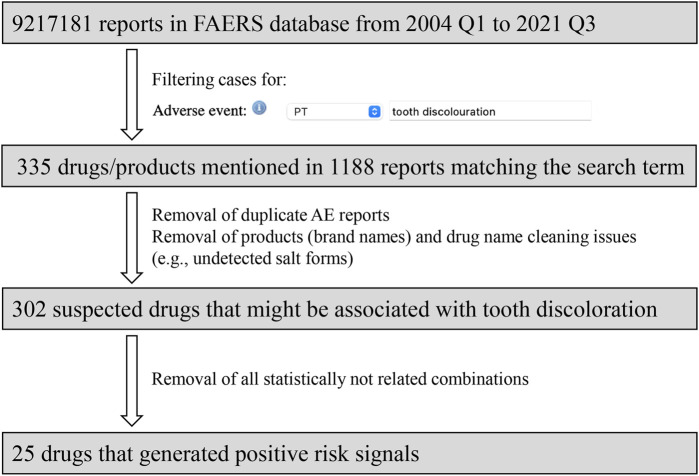
Flow diagram of drug identification in the FAERS.

Herein, the MedDRA PT: “tooth discoloration” was employed to identify the relevant cases. Possible duplicate and multiple records were then examined and excluded. For each reported suspected drug, all possible drug names were taken into account, and the same active substance was combined by generic name. Anatomical therapeutic chemical (ATC) codes were assigned to each drug, in order to group all tooth discoloration cases for each active substance. The flow diagram of data mining is presented in Figure 1.

### Statistical analysis

Disproportionality analyses were performed in this study. Two statistical metrics, proportional reporting ratio (PRR) and reporting odds ratio (ROR), were employed to characterize the association between the drug of interest and tooth discoloration.

Each analysis of the potential association between drug exposure and AE utilized a two-by-two contingency table (Table 1), through which disproportional AEs and drug combinations could be identified. The PRR is a disproportionate degree of adverse event reporting for a particular drug compared to the reports of the same adverse event for all other drugs in the database. Based on criteria presented by Evans and collaborators (Evans et al., 2001), a positive signal was commonly considered for PRR≥2, chi-square value ≥ 4, and ≥3 cases. The value of PRR is positively correlated to the strength of the association. The ROR is the ratio of the reporting rate of a particular event to the totality of other events for a particular drug to that of the remaining drugs included in the database. Signals were detected with the lower bound of the 95% two-sided confidence interval (CI) for the ROR above 1 (van Puijenbroek et al., 2002). The higher the value, the more pronounced the disproportionate performance.

**TABLE 1:** Two-by-two contingency table for disproportionality analyses.

	Adverse events of interest	All other adverse events of interest	Total
Drug of interest	a	b	a + b
All other drugs of interest	c	d	c + d
Total	a + c	b + d	a + b + c + d

Proportional reporting ratio (PRR): $PRR=\frac{a/\left(a+b\right)}{c/\left(c+d\right)},95\%CI=e^{\ln\left(PRR\right)\pm 1.96\sqrt{\frac{1}{a}-\frac{1}{a+b}+\frac{1}{c}-\frac{1}{c+d}}}$

Reporting odds ratio (ROR): $ROR=\frac{a/c}{b/d},95\%CI=e^{\ln\left(ROR\right)\pm 1.96\sqrt{\frac{1}{a}+\frac{1}{b}+\frac{1}{c}+\frac{1}{d}}}$

To decrease the false-positive rate of risk signals, the threshold of detection criteria was improved in this study. For PRR, a positive signal was defined by PRR≥3, chi-square value ≥ 5, and ≥5 cases. For ROR, positive signals were identified with the low boundary of 95% CI > 2 and at least 5 cases submitted (Chen et al., 2022).

## Results

Through OpenVigil 2.1, 9,217,181 AE reports were extracted between 2004 Q1 and 2021 Q3. Based on predefined inclusion criteria, 1188 AE reports involving 302 suspected drugs were identified. Table 2 describes the characteristics of AE reports regarding tooth discoloration. With respect to gender, most patients (59.3%) were males. The patients aged 45–64, ≤18, and 19–44 accounted for 17.0%, 11.9%, and 10.2%, respectively. Most reports (76.5%) were submitted from the United States.

**TABLE 2:** The characteristics of AE reports pertaining to tooth discoloration.

Characteristics	N (%)
Patient gender	
Male	705 (59.3%)
Female	334 (28.1%)
Unknown or missing	149 (12.5%)
Patient age group (years)	
≤18	141 (11.9%)
19–44	121 (10.2%)
45–64	202 (17.0%)
65–74	68 (5.7%)
≥75	65 (5.5%)
Unknown or missing	591 (49.7%)
Reporting country	
United States	909 (76.5%)
United Kingdom	66 (5.6%)
Canada	15 (1.3%)
German	15 (1.3%)
Others	91 (7.7%)
Unknown or missing	92 (7.7%)
Reporting Region	
America	930 (78.3%)
Europe	136 (11.5%)
Asia	20 (1.7%)
Oceania	9 (0.8%)
Africa	1 (0.1%)
Unknown or missing	92 (7.7%)

Disproportionality analyses based on two algorithms were performed, and the results are presented in Table 3. After data mining, 25 drugs had positive risk signals for tooth discoloration. The most commonly reported drug associated with tooth discoloration was tetracycline (*n* = 106), followed by salmeterol and fluticasone (*n* = 68), amoxicillin (*n* = 60), chlorhexidine (*n* = 54), and nicotine (*n* = 52). Of all the 25 drugs examined, cetylpyridinium (PRR = 472.2, ROR = 502.5), tetracycline (PRR = 220.4, ROR = 277), stannous fluoride (PRR = 254.3, ROR = 262.8), hydrogen peroxide (PRR = 240.0, ROR = 247.6), and chlorhexidine (PRR = 107.0, ROR = 108.4) showed stronger associations with tooth discoloration than the remaining drugs.

**TABLE 3:** Drugs associated with tooth discoloration based on the FAERS.

Drugs	N	χ^2^	PRR (95% CI)	ROR (95% CI)
Tetracycline	106	25,472.3	268.3 (220.4–326.6)	277.0 (226.3–339.2)
Salmeterol and fluticasone	68	191.0	4.8 (3.8–6.2)	4.8 (3.8–6.2)
Amoxicillin	60	478.6	10.5 (8.1–13.6)	10.5 (8.1–13.6)
Chlorhexidine	54	5310.3	107.0 (81.5–140.3)	108.4 (82.3–142.6)
Nicotine	52	364.7	9.4 (7.1–12.4)	9.4 (7.1–12.4)
Asenapine	34	1275.1	41.8 (29.7–58.7)	42.0 (29.8–59.1)
Minocycline	31	998.6	36.2 (25.3–51.6)	36.3 (25.4–51.9)
Linezolid	26	462.2	20.9 (14.2–30.8)	20.9 (14.2–30.9)
Budesonide	25	39.4	3.4 (2.3–5.1)	3.4 (2.3–5.1)
Doxycycline	23	148.2	8.8 (5.8–13.3)	8.8 (5.8–13.3)
Zoledronic acid	23	49.4	4.1 (2.7–6.2)	4.1 (2.7–6.2)
Alendronic acid	19	29.3	3.4 (2.2–5.3)	3.4 (2.2–5.3)
Sucroferric oxyhydroxide	18	831.3	51.6 (32.5–82.1)	52.0 (32.6–82.9)
Azithromycin	17	83.7	7.1 (4.5–11.6)	7.2 (4.5–11.7)
Clarithromycin	15	59.3	6.2 (3.7–10.3)	6.2 (3.7–10.3)
Sodium fluoride	13	886.2	76.6 (44.5–132.0)	77.4 (44.7–134.0)
Hydrogen peroxide	10	2129.3	240.0 (130.0–443.0)	247.6 (131.6–465.8)
Colestyramine	9	153.6	21.3 (11.1–41.1)	21.4 (11.1–41.3)
Cetylpyridinium	9	3745.7	472.2 (250.0–891.8)	502.5 (255.5–988.3)
Ertapenem	9	255.2	34.1 (17.7–65.6)	34.3 (17.8–66.1)
Rifampicin	9	39.0	6.9 (3.6–13.2)	6.9 (3.6–13.2)
Stannous fluoride	6	1264.7	254.3 (115.5–559.8)	262.8 (116.3–594.1)
Triclosan	6	430.3	87.9 (39.6–195.3)	88.9 (39.7–199.3)
Cefprozil	5	299.8	76.5 (31.9–183.4)	77.3 (32.0–186.8)
Pamidronic acid	5	21.8	7.5 (3.1–18.0)	7.5 (3.1–18.0)

PRR, proportional reporting ratio; ROR, reporting odds ratio; CI, confidence interval.

The characteristics of 652 AE reports involving 25 drugs with positive risk signals were presented in Table 4. We stratified dosage, patient age, and outcomes based on these 25 drugs. Most of them could be prescribed in appropriate dosage ranges except two cases of asenapine exceeding the recommended dosage. 49.8% (325/652) of the AE reports did not record patient age. Of the remaining 327 AE reports, tooth discoloration was mostly reported in patients aged 45–64 (*n* = 110) and ≤18 (*n* = 95). Since the labels of tetracyclines indicated that tooth development involved the second and third trimesters of pregnancy, infancy, and childhood up to the age of 8 years, we also paid attention to drug administration in patients aged under eight. In antimicrobial-related cases, some patients were younger than 8 years, such as amoxicillin (*n* = 28), tetracycline (*n* = 6), doxycycline (*n* = 3), azithromycin (*n* = 3), linezolid (*n* = 2), clarithromycin (*n* = 2), and cefprozil (*n* = 2). Also, there were nine and two cases involving patients aged under eight for budesonide and salmeterol and fluticasone, respectively. 29.3% (191/652) of the cases reported serious outcomes, which were documented as other (*n* = 161), hospitalization-initial or prolonged (n = 38), disability (*n* = 30), required intervention to prevent permanent impairment (*n* = 6), damage (*n* = 6), death (*n* = 4), life-threatening (*n* = 2), and congenital anomaly (*n* = 2). Of note, one AE report might record more than one serious outcome.

**TABLE 4:** The characteristics of AE reports involving drugs with positive risk signals of tooth discoloration.

Drugs	N	Dosage (N)	Patient age group (years)						Serious Outcome^a^
			≤18	19–44	45–64	65–74	≥75	Unknown	
Tetracycline	106	20 mg/day (2)	7	6	18	1	0	74	12
		1000 mg/day (1)							
		Unknown (103)							
Salmeterol and fluticasone	68	1 puff/day (3)	8	1	8	8	3	40	10
		2 puffs/day (25)							
		Unknown (40)							
Amoxicillin	60	8–28 mL/day (25)	36	5	1	4	1	13	25
		1750–4,000 mg/day (3)							
		1–3 DF/day (9)							
		Unknown (23)							
Chlorhexidine	54	Unknown (54)	0	1	4	2	2	45	3
Nicotine	52	2 mg/day (5)	0	9	14	9	4	16	7
		4 mg/day (8)							
		Unknown (39)							
Asenapine	34	5 mg/day (1)	0	6	6	1	0	21	1
		10 mg/day (8)							
		15 mg/day (1)							
		20 mg/day (7)							
		25 mg/day (1)							
		30 mg/day (1)							
		Unknown (15)							
Minocycline	31	4 mg/day subgingival (2)	4	2	17	0	0	8	15
		100 mg/day (1)							
		200 mg/day (5)							
		300 mg/day (3)							
		Unknown (20)							
Linezolid	26	90 mg/kg/day (1)	6	6	4	1	1	8	6
		100 mg/day (1)							
		600 mg/day (2)							
		1200 mg/day (13)							
		Unknown (9)							
Budesonide	25	0.5 mg/day (1)	12	1	2	2	1	7	7
		1 mg/day (1)							
		160 μg/day (1)							
		180 μg/day (1)							
		320 μg/day (1)							
		360 μg/day (1)							
		400 μg/day (1)							
		640 μg/day (1)							
		Unknown (17)							
Doxycycline	23	40 mg/day (1)	4	1	6	1	2	9	9
		100 mg/day (2)							
		200 mg/day (2)							
		250 mg/day (1)							
		Unknown (17)							
Zoledronic acid	23	3 mg/day (1)	0	1	5	1	1	15	22
		4 mg/day (9)							
		5 mg/day (2)							
		Unknown (11)							
Alendronic acid	19	70 mg, qw (5)	1	2	7	1	4	4	18
		Unknown (14)							
Sucroferric oxyhydroxide	18	2250 mg/day (2)	0	1	3	0	0	14	0
		Unknown (16)							
Azithromycin	17	4 mL/day (1)	9	1	2	0	0	5	12
		120 mg/day (1)							
		200 mg/day (2)							
		Unknown (13)							
Clarithromycin	15	1000 mg/day (4)	4	2	2	1	3	3	8
		Unknown (11)							
Sodium fluoride	13	Unknown (13)	1	1	1	0	1	9	3
Hydrogen peroxide	10	Unknown (10)	0	2	4	0	0	4	10
Colestyramine	9	4 g/day (4)	0	0	0	0	2	7	0
		8 g/day (3)							
		9 g/day (1)							
		Unknown (1)							
Cetylpyridinium	9	20 mL/day (2)	0	5	1	2	0	1	5
		Unknown (7)							
Ertapenem	9	1g/day (3)	0	0	1	2	1	5	6
		Unknown (6)							
Rifampicin	9	150 mg/day (5)	0	1	4	1	1	2	5
		450 mg/day (1)							
		Unknown (3)							
Stannous fluoride	6	20 mL (2)	0	1	0	2	0	3	1
		Unknown (4)							
Triclosan	6	Unknown (6)	0	0	0	0	0	6	0
Cefprozil	5	Unknown (5)	3	0	0	0	0	2	1
Pamidronic acid	5	90 mg, qmon (2)	0	1	0	0	0	4	5
		Unknown (3)							

DF, dosage form.

^a^ Serious outcome includes death, hospitalization-initial or prolonged, life-threatening, disability, congenital anomalies, and/or other.

**FIGURE 2:**
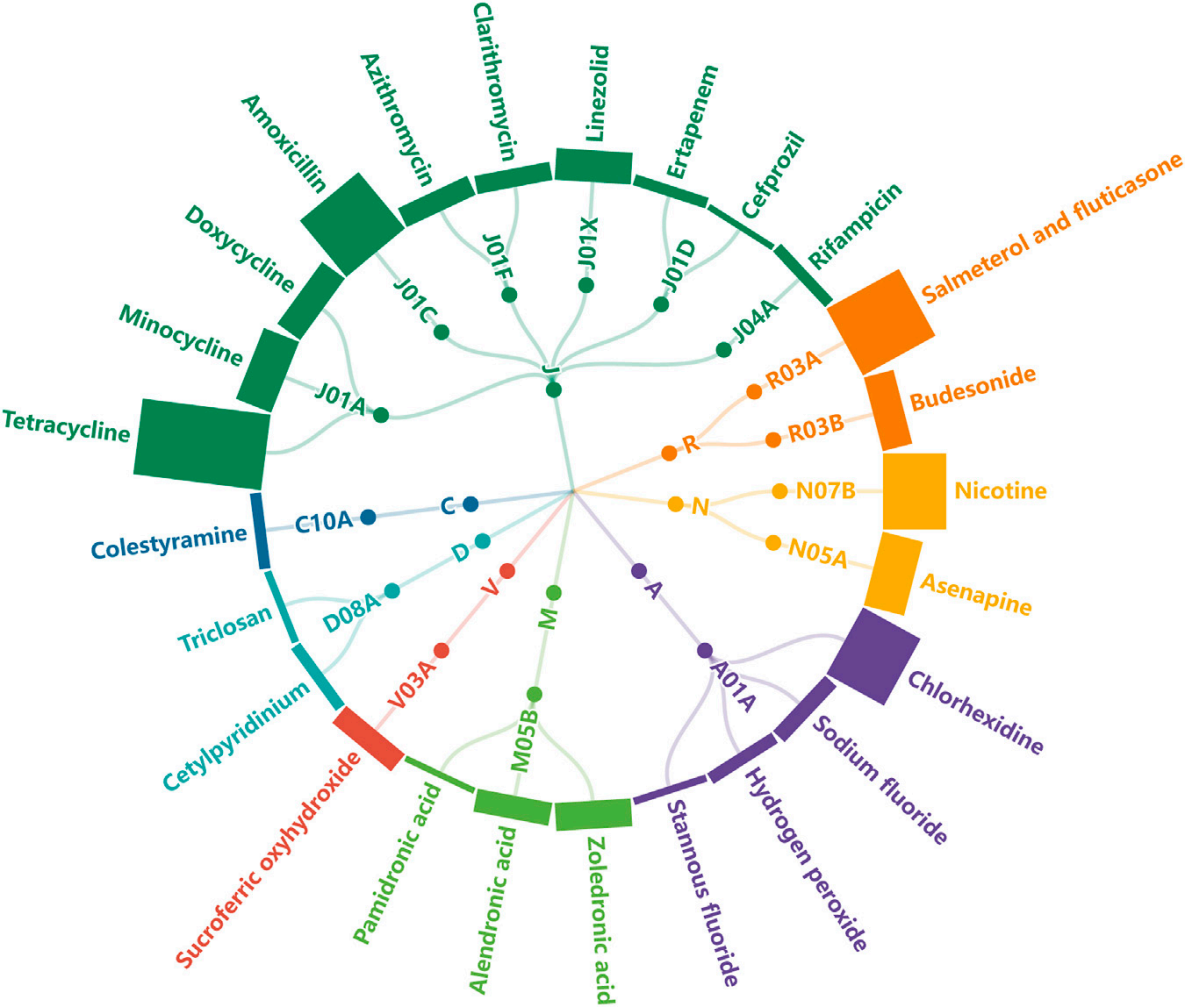
Radical tree of drugs grouped by ATC class. The height of each column represents the number of submitted AE reports (J: anti-infectives for systemic use, R: respiratory system, N: nervous system, A: alimentary tract and metabolism, M: musculo-skeletal system, V: various, D: dermatologicals, C: cardiovascular system).

The distribution of the 25 drugs based on ATC code is exhibited in Figure 2, in which the column height represents the number of corresponding AE reports. The involved ATC classes included anti-infectives for systemic use (*n* = 10), alimentary tract and metabolism *n* = 4), musculo-skeletal system (*n* = 3), respiratory system (n = 2), dermatologicals (*n* = 2), nervous system (*n* = 2), cardiovascular system (*n* = 1), and various (*n* = 1).

## Discussion

This represents the first report to comprehensively review drugs associated with tooth discoloration. Based on FAERS, 1188 AE reports were submitted in the study period. Totally 25 drugs were statistically associated with tooth discoloration, involving eight ATC classes. These drugs could be divided into two categories according to administration route, including 8 drugs for topical use and 17 for systemic use.

### Drugs for topical use

#### Stomatological preparations

Stannous fluoride and sodium fluoride are fluorides, a class of compounds containing fluoride. Although fluoride-containing products promote tooth resistance to caries, long-term use of fluoride has adverse effects on the tooth. During enamel formation and maturation, tooth discoloration might be caused by high total daily intake of fluoride ions. Dental fluorosis constitutes a less severe form of chronic toxicity due to chronic intake of high fluoride amounts during tooth development and eruption (Abanto Alvarez et al., 2009). It features enamel hypomineralization, with white flecks mainly detected on cusp tips and facial surfaces of permanent teeth. In moderate-to-severe forms, extended brown stains and cavities are present on most permanent teeth. Dental fluorosis correlates with condition dose, and the higher the exposure level during tooth development, the severer the disorder. A four-year randomized controlled trial demonstrated markedly elevated tooth discoloration incidence in the intervention group compared with control patients not using any fluoride product (Frese et al., 2019).

Chlorhexidine as a broad-spectrum antiseptic has a well-documented dental plaque-inhibiting effect. Its mechanism of action is that chlorhexidine (positive charge) interacts with the microbial cell wall (negative charge), destabilizing the microorganism’s osmotic equilibrium. Mouthwashes containing chlorhexidine are mainly indicated for utilization as adjuncts to mechanical cleaning, whereas long-term application induces several local adverse effects, prominently including the generation of brown stains on teeth and oral tissues (James et al., 2017). It is admitted stains mostly result from the precipitation of anionic dietary chromogens, including tea, coffee, and wine constituents, onto the adsorbed chlorhexidine cations, so the mechanism by which chlorhexidine causes extrinsic tooth staining seems to be highly related to its mechanism of action (Watts and Addy, 2001).

Hydrogen peroxide is a strong oxidizing agent, which has been widely used in dentistry to bleach teeth for more than 70 years. Hydrogen peroxide generates the perhydroxyl free radical HO_2_^−^, which has high reactivity and substantial oxidative potential, oxidizing tooth discoloration. Hydrogen peroxide can cause damage to the enamel of the teeth if used incorrectly. High hydrogen peroxide amounts can damage the integrity of the enamel surface, and teeth may show higher susceptibility to extrinsic discoloration following bleaching because of elevated surface roughness (Tredwin et al., 2006).

#### Drugs for obstructive airway diseases

Inhalation therapy is the mainstay of drug therapy for obstructive airway diseases. In this study, therapeutic drugs including salmeterol and fluticasone and budesonide generated positive risk signals for tooth discoloration. However, 10 patients with tooth discoloration were under 8 years old, which should be paid more attention to. Though few studies focused on inhalation-induced tooth discoloration, the latter might be attributed to tooth erosion via alteration of the mouth’s chemical environment (Thomas et al., 2010). Erosion, abrasion, and attrition result in a gradual impairment of enamel and dentine, and manifest as tooth wear. As enamel thinning occurs, the tooth becomes darker with dentine color becoming more obvious (Watts and Addy, 2001). Inhalants expose patients to the risk of tooth erosion by decreasing the protective effects of saliva on external or intrinsic acids (Thomas et al., 2010). Saliva is a major factor neutralizing daily dietary acids in the oral cavity. Studies showed a reduced salivary flow rate in asthmatics administered beta-2 adrenoceptor agonists in comparison with non-asthmatic subjects. Reduced saliva flow or quality can affect proteins and chemical composition in the salivary pellicle, which plays a role in protecting teeth from erosive wear (Goswami et al., 2021). Decreased intraoral pH after the use of inhalers may also be associated with tooth erosion. O’Sullivan and colleagues reported anti-asthmatics, especially powdered products (beclomethasone diproprionate, fluticasone, salmeterol, and terbutaline sulfate powders), could induce a pH below the value of 5.5 that is required to dissolve hydroxyapatite (O'Sullivan and Curzon, 1998). A decrease in salivary pH after using an inhaler several times a day, particularly lactose-based inhalers, possibly causes tooth dissolution. Gastroesophageal reflux disease may be another risk factor for tooth erosion. Long-acting beta-agonists relax smooth muscles and potentially facilitate acid reflux (McCallister et al., 2011).

#### Antiseptics and disinfectants

Cetylpyridinium chloride, a quaternary ammonium compound, efficiently enhances the antimicrobial effects of oral hygiene products. It is widely used in over-the-counter products such as mouthwashes and toothpastes (Mao et al., 2020). As a cationic detergent, its interactions with cell membranes can lead to the leakage of cell components, disrupt bacterial metabolism, inhibit cell growth, and induce cell death.

Triclosan has been used since the 1960s as a broad-spectrum antibacterial agent and is added to a variety of consumer products such as soaps, hand sanitizers, toothpastes, mouthwashes, and cosmetics (Weatherly and Gosse, 2017). Triclosan has been incorporated into toothpaste with sodium fluoride to enhance inhibitory effects on bacterial metabolism in dental plaques. In this study, all six reported cases related to triclosan concomitantly used sodium fluoride.

### Drugs for systemic use

#### Anti-infectives

Since 1948, tetracyclines have been utilized to treat a variety of infections, especially *brucella* infections for which they are the antibiotics of choice. In 1956, tetracycline was firstly reported to cause discoloration of children’s teeth (Schuster and Shwachman, 1956), and many subsequent reports have indicated that tetracycline also causes tooth enamel hypoplasia. Tetracycline irreversibly binds to calcified structures in teeth when utilized during the calcification phase of tooth development, forming a visible discoloration when the tetracycline layer of the tooth is oxidized by light. The discoloration extent depends on the type of tetracyclines, dosage, treatment time, and patient age at treatment. Deciduous teeth begin to calcify approximately at the end of pregnancy month four. The calcification of permanent teeth begins after birth and is completed at the age of seven-eight years. Therefore, administration of tetracyclines must be avoided in the second and third trimesters of gestation and pediatric patients below 8 years (Sánchez et al., 2004). In our study, six patients younger than 8 years old developed tooth discoloration after treating with tetracycline. Previous data showed that the incidence rates of tooth discoloration caused by different tetracyclines vary between 23% and 92% in children (Todd et al., 2015). Tooth discoloration was also found in adults after long-term use of tetracyclines (Chiappinelli and Walton, 1992).

Minocycline, a semisynthetic tetracycline antibiotic, is frequently applied to treat acne vulgaris and rosacea, and utilized as an adjunct treatment of periodontal disease. Minocycline causes tooth discoloration, with an incidence of 3%–6% in adults taking minocycline more than 100 mg/d for long-term use (Good and Hussey, 2003). Minocycline also causes abnormal pigmentation of skin, thyroid gland, nails, bones, sclera, and conjunctiva in adult individuals (Ran et al., 2021).

Doxycycline is a tetracycline derivative firstly marketed in 1967. Considering tooth staining, the FDA requires all tetracycline drugs must bear a warning label of not being suitable for children under 8 years unless no other effective antibiotic agents are available. Compared with other tetracyclines, doxycycline has a lower affinity with calcium, and therefore, the risk of tooth staining is relatively lower. Six reports investigated tooth discoloration in ≥338 patients administered doxycycline below 8 years old (Stultz and Eiland, 2019). In the latter studies, tooth discoloration was similar between treated and control patients, indicating doxycycline is a safer alternative in case tetracyclines are required in patients under eight. The recommendation from the American Academy of Pediatrics points out that only doxycycline may be utilized for short treatments (<21 days) in all age groups (Stultz and Eiland, 2019). However, it should be noted there are still some case reports of doxycycline-induced discoloration of the permanent dentition (Ayaslioglu et al., 2005; Nelson and Parker, 2006). Herein, 23 AE reports of tooth discoloration involved doxycycline, of which three reports involved patients aged under eight.

Though not exerting a positive signal, tigecycline might be associated with tooth discoloration. Tigecycline could induce permanent discoloration of the teeth if administered by injection during tooth development (second half of pregnancy, infancy, and childhood before 8 years). A retrospective study evaluated the discoloration of permanent teeth in children below 8 years after tigecycline exposure. The study eventually included 12 patients, of whom two (16.7%) developed yellow discoloration of the tooth (Zhu et al., 2021).

Amoxicillin is one of the most widely used penicillins, designated by the WHO as a “core access antibiotic.” Amoxicillin and amoxicillin/clavulanic acid are usually well tolerated. Amoxicillin/clavulanate appeared to increase the risk of gastrointestinal adverse events, Stevens-Johnson syndrome, purpura, and hepatitis compared with amoxicillin monotherapy (Salvo et al., 2007). In addition, a higher proportion of stomatological reactions were reported with amoxicillin/clavulanic acid. The AEs associated with oropharyngeal lesions (enanthema, gingivitis, stomatitis, tongue disorder, and tooth discoloration) occurred later compared with other AEs after drug administration. These stomatological reactions may be attributed to the alteration of the bacterial flora due to the elimination of sensitive micro-organisms, as a result of long-term treatment. Garcia-López and collaborators (Garcia-López et al., 2001) reported three pediatric tooth discoloration cases who took amoxicillin/clavulanic acid. The drug surveillance programs of Australia and the Netherlands also reported this AE, as well as a study using the data from the Spanish Regional Drug Surveillance Centre. Herein, we also found 28 patients aged below eight developed tooth discoloration after using amoxicillin.

Carbapenems are *β*-lactam antibacterial drugs with a very broad spectrum. To date, the most commonly applied carbapenems include meropenem, imipenem, and ertapenem. Ertapenem, a broad-spectrum agent with limited effects on non-fermentative Gram-negative bacilli, is most indicated for community-acquired infections (Keating and Perry, 2005). Tooth staining has been identified during post-approval use of ertapenem. Dry mouth is one of its gastrointestinal adverse reactions, which may result in tooth discoloration due to decreased saliva secretion.

Macrolides represent a diverse group of hydrophobic molecules comprising macrolide rings and variable side chains or groups that inhibit protein synthesis by targeting bacterial ribosomes. Commonly used macrolides in clinic include azithromycin, erythromycin, and clarithromycin. Gastrointestinal reactions represent the most common side effects of macrolides. During a 12-week course of treatment with clarithromycin and rifabutin, some pediatric patients had gastrointestinal complaints, fever, and tooth discoloration (Lindeboom, 2011). In the current study, tooth discoloration was also reported in three patients aged below eight after application of azithromycin, as well as two patients aged below eight treated with clarithromycin. Fortunately, tooth discoloration induced by macrolides was usually reversed by professional cleaning upon discontinuation of these drugs.

Linezolid is the first marketed antibiotic of the oxazolidinone class with demonstrated activity against a variety of Gram-positive bacteria. From a safety perspective, linezolid is generally safe and well-tolerated when used for short courses of treatment. Several mild-to-moderate adverse reactions have been reported, including myelosuppression, neuropathy (peripheral and optic), gastrointestinal effects, elevated liver enzymes, skin eruption, and lactic acidosis. Tooth discoloration as a rare side effect mostly occurred in children. Matson and Miller (Matson and Miller, 2003) described an 11-year-old female showing tooth discoloration in a 28-day oral treatment with linezolid (600 mg, bid). Ma (Ma, 2009) reported an eight-year-old female with brownish discoloration of teeth and tongue after orally taking 1 week of linezolid (30 mg/kg/day in three doses). Petropoulou and colleagues (Petropoulou et al., 2013) described three children with similar side effects during intravenous treatment with linezolid (30 mg/kg/day, q8h). Santos *et al.* (Santos et al., 2015) reported a nine-year-old female showing linear brownish enamel discoloration on both the upper and lower anterior teeth 4 weeks upon initiation of oral linezolid (30 mg/kg/day, q8h). Agrawal et al. (2018) reported dental hyperpigmentation in an adult who presented with discoloration of both the upper and lower teeth after treatment with linezolid (600 mg, bid) for 2 months. Zou et al. (2020) reported tooth discoloration induced by intravenous linezolid (10 mg/kg, q8h for 12 days) in children in Mainland China. Likewise, we also found two patients aged below eight with tooth discoloration after using linezolid. Tooth discoloration caused by linezolid is extrinsic and reversible, which can be removed by extensive dental cleaning (Kadam, 2008). For oral administration, tooth discoloration may be attributed to the direct exposure of teeth and tongue to linezolid. However, for intravenous administration, tooth discoloration may be associated with changes of the normal flora in the oral cavity (Santos et al., 2015). The incidence of tooth discoloration is higher in children than in adults, which may be attributed to the discrepancy in oral microbial communities between adults and children (Zou et al., 2020). Pediatric patients and their parents should be informed of this potential reversible side effect prior to linezolid treatment.

Rifampicin was firstly introduced in 1968 and remains a major anti-tuberculous drug (Abulfathi et al., 2019). It is well known that rifampicin can cause discoloration (yellow, orange, red, or brown) of body fluids (urine, sweat, sputum, and tears), as well as tooth discoloration, which may be permanent.

#### Nervous system agents

As mentioned above, since saliva helps remove food particles and forms a biofilm on teeth, the reduction of salivary secretion may lead to extrinsic tooth staining. The salivary gland secretion primarily involves the parasympathetic nervous system, as well as the sympathetic nervous system. Medications blocking the nervous system can result in decreased salivation, which is indirectly associated with tooth discoloration (Tredwin et al., 2005). Our findings showed that the neurologic drug asenapine was associated with tooth discoloration, with an ROR of 41.983, which may be related to sublingual administration. Nicotine in tobacco, although colorless, turns yellow after oxidation and sticks to the teeth, causing tooth discoloration. Nicotine replacement treatment is broadly applied to effectively address tobacco dependence. All licensed forms (gum, transdermal patch, nasal spray, inhalator, and sublingual tablet/lozenge) can help elevate the chances of successful cessation in people who attempt to quit smoking. Of 52 nicotine-related cases, 12 were buccal route, 2 were inhaled, and 3 were oral. *In vitro* tests have shown that nicotine polacrilex gum utilized to treat smoking cessation may exert stain-reducing (i.e., whitening) effects on smokers’ teeth. In a randomized controlled trial, a nicotine replacement gum removed stains and whitened teeth better than a nicotine replacement sub-lingual tablet during a six-week smoking cessation program. Smoking cessation interventions combined with stain removal or tooth whitening effects might further motivate to quit, and oral healthcare givers could provide patients with smoking cessation advices and support (Whelton et al., 2012).

#### Drugs affecting bone structure and mineralization

Bisphosphonates constitute a drug group preventing bone density reduction, which are commonly utilized for the treatment of osteoporosis and related disorders. Structurally, bisphosphonates are derivatives of inorganic pyrophosphate. Two groups of bisphosphonates are known, i.e., non-nitrogen-containing (e.g., etidronate, clodronate, and tiludronate, considered first-generation bisphosphonates) and nitrogenous (e.g., alendronate, pamidronate, and zoledronic acid, considered second- and third-generation bisphosphonates) molecules. Bisphosphonates have very high affinities for bone minerals because of their interaction with hydroxyapatite crystals, inhibiting osteoclast activity and thereby effectively suppressing bone resorption. Hydroxyapatite is the major mineral component of vertebrate bones and teeth. The enamel and dentin of human teeth are composed of 97% and 70% inorganic components, respectively. These inorganic phases are mainly composed of hydroxyapatite (Chen et al., 2021). The shape and size of hydroxyapatite crystallites in dentin are very similar to those in bone. Therefore, it is speculated that bisphosphonates may cause tooth discoloration by affecting hydroxyapatite. Additionally, due to the local effects of oral bisphosphonates on the esophagus and/or gastric mucosa, upper gastrointestinal adverse effects, e.g., reflux, esophagitis, and esophageal ulcer, are frequent causes of intolerance to oral bisphosphonates. Of note, reflux may affect tooth color by lowering the pH of the oral microenvironment and eroding hydroxyapatite in enamel and dentin.

#### Other therapeutic drugs

Drugs containing metallic compounds can lead to extrinsic staining of teeth. Addy et al. (1985) reported that certain metals (especially iron and tin) can cause tooth discoloration. For example, dark brown to black discoloration was observed in people taking iron supplements, and oral iron salts in liquid form could cause the teeth to appear greenish black (Teoh et al., 2019). Sucroferric oxyhydroxide represents a phosphate binder that effectively controls serum phosphorus amounts in chronic kidney disease cases on dialysis and is mainly supplied as chewable tablets for oral use in a brown color. Tooth discoloration was reported during its post-marketing use.

The FAERS database is a great tool with sufficient reports to identify rare adverse reactions that are difficult to detect in conventional epidemiologic studies. However, some inherent limitations still exist. A major limitation is the high risk of selection and reporting biases. The self-reported data in FAERS do not accurately estimate the actual volume of AEs to the drugs, since they are inherently limited by underreporting of cases. Therefore, the FAERS may not be utilized to estimate the true incidence of a given AE for the drug. Another limitation is the data quality issues in terms of duplicate reports and missing information (such as age, dosage, and treatment course), which affect further analyses of these data. It also should be noted that the positive risk signals obtained from the analysis can only demonstrate associations and not causality. In other words, whether the suspected drug mentioned in the report can lead to the AE of interest is uncertain. Further epidemiologic studies are still warranted to clarify such associations in the appropriate clinical setting.

## Conclusion

This study assessed drug-related tooth discoloration by analyzing AEs reported in the FAERS database. We found 25 drugs were associated with tooth discoloration, among which anti-infectives, stomatological preparations, and drugs affecting bone structure and mineralization accounted for the majority. Together with previous investigations, our data provide further evidence that certain drugs may cause tooth discoloration. Caution should be exercised when using these drugs, particularly during pregnancy and early childhood. In addition, patients should be instructed to pay special attention to oral hygiene measures. Further investigation is still needed to confirm these findings and unveil the underpinning mechanism of tooth discoloration.

## Data Availability

The raw data supporting the conclusion of this article will be made available by the authors, without undue reservation.
